# Developing a Depression Care Model for the Hill Tribes: A Family- and Community-Based Participatory Research

**DOI:** 10.1155/2023/3191915

**Published:** 2023-10-12

**Authors:** Onnalin Singkhorn, Pawadee Hamtanon, Katemanee Moonpanane, Khanittha Pitchalard, Rachanee Sunsern, Yosapon Leaungsomnapa, Chananan Phokhwang

**Affiliations:** ^1^School of Nursing, Mae Fah Luang University, Chiang Rai Province, Thailand; ^2^Center of Excellence for the Hill Tribe Health Research and Training, Mae Fah Luang University, Chiang Rai, Thailand; ^3^Nursing Faculty, Thaksin University, Phatthalung, Thailand; ^4^School of Health science, Mae Fah Luang University, Thailand; ^5^Phrapokklao Nursing College, Chanthaburi, Faculty of Nursing, Praboromarajchanok Institute, Ministry of Public Health, Thailand; ^6^Nursing Faculty, Rajabhat University, Surat Thani, Thailand

## Abstract

A high prevalence of depression has been detected among individuals from the hill tribes in Thailand. However, there are no proper interventions to address this problem. Using a community-based participatory research (CBPR) design, the study team developed a model of depression care for this population. The study involved 45 people in the model development and 65 people in the model testing, who were patients, family members, village health volunteers (VHVs), community and religious leaders, healthcare personnel, NGOs, and local administrative staff. The model development was divided into three phases: understanding the current situation of depression and care, model development, and evaluation of its effectiveness using psychological and relevant outcomes. Questionnaires, observations, focus groups, and in-depth interviews were used for data collection, and content analysis was employed for qualitative data. The Wilcoxon signed-rank test was used to analyze changes in VHVs' knowledge and skills before and after training. The resulting model, “SMILE,” consists of stakeholders' readiness (S), external and internal motivations (M), interpersonal relationship (I), life and community assets (L), and empowerment (E). VHVs underwent training on the model, and after training, their knowledge increased significantly from 3.50 ± 1.14 to 8.28 ± 0.81 (*p* < 0.001). Moreover, their basic counselling and depression screening skills showed improvement from 3.39 ± 1.23 to 7.64 ± 3.76 (*p* < 0.001). The developed model can be applied to other hill tribe communities in Northern Thailand to improve depression care.

## 1. Introduction

Depression is a global mental health problem. Approximately 280 million people worldwide suffer from depression, which causes illness and disability [1]. In 2017, approximately 5.7% of these were older adults. Depression is a significant cause of suicide; roughly 800,000 people with depression commit suicide each year [2]. Previous studies have identified factors associated with depression, such as physical health illness [3], ethnicity [4], substance abuse [5], history of sexual abuse [6], marital status, stress events [5, 7], and gender [5, 8]. Regarding ethnicity-related depression, the results of a cross-sectional study with 601 people from a hill tribe in Chiang Rai Province, Thailand, aged 40 years and older, showed that approximately 39.1% had depression. Of these, 64.2% were female, and 35.8% were male. In addition, approximately 75.7%, 17.9%, and 6.4% had mild, moderate, and severe depression, respectively. Being female and having no education, having low income, and having physical illness contributed to depression [5].

According to the review literature, no previous study showed a specific model based on family and community participation to care for patients with mental illnesses in Thailand, especially those with ethnic backgrounds, such as the hill tribes, where societal disparities were immense. The WHO estimated that Thailand had approximately 3.5-4 million people from hill tribes in 2019 [9]. However, approximately 1 million from six majority ethnic groups (Lisu, Mien, Karen, Hmong, Akha, and Lahu) lived in the mountain borders of six northern provinces (Chiang Rai, Chiang Mai, Mae Hong Son, Lamphun, Nan, and Tag) between Myanmar, Thailand, and Laos [10]. Barriers to healthcare services for the hill tribes include cultural practice and beliefs, insufficient income, low education, language, road-travel inaccessibility, no Thai identification documents, societal inferiority, and illegal methamphetamine and opium use-related stigma. Developing a depression care model and caring for people with depression require cooperation between mental healthcare providers, public health providers, academic institutions, local administration organizations, families, and the community. In this community-based participatory research (CBPR), an academic-community partnership study, we developed a depression care model based on WHO's Innovation Care for Chronic Conditions (ICCC) framework [11] for people from hill tribes with depression.

The chosen theoretical framework, the ICCC, has inspired various interventions and has been applied in several countries with diverse healthcare systems and socioeconomic contexts [12]. Numerous studies have demonstrated its suitability and effectiveness for patients with chronic illnesses in the community, including patients with diabetes mellitus (DM) [13] and severe mental disorders [14–16]. It is applicable to both chronic physical and mental illnesses due to commonalities in their determinants, consequences, and shared healthcare strategies for prevention and management [17]. In the context of the ICCC application, some nurse case managers in Canada have successfully applied this framework to care for elderly chronic patients, emphasizing community involvement in assessing, planning, and providing care [15]. Furthermore, CBPR is action-oriented and aimed at supporting and enhancing strategic actions for community transformation and social change [18]. It is particularly suited to addressing healthcare challenges among culturally vulnerable and underserved populations [19]. A systematic review and meta-analysis have indicated that community-based psychosocial interventions for individuals with schizophrenia lead to positive outcomes in terms of functionality and reduced rehospitalization rates [20].

Therefore, the present study is aimed at describing the development of a depression care model for individuals from hill tribes dealing with depression, based on the aforementioned theories.

## 2. Methods

### 2.1. Design

In reporting action research of this kind, we conformed as much as possible to the Consolidated Criteria for Reporting Qualitative Research (COREQ).

We started this family-community-participation project by surveying the depression care system, current problems, and needs of patients with depression, their relatives, and the community from the Ban Lao-Fu Village of the Pa-Tung subdistrict, Maejan District, Chiang Rai Province, where more than 300,000 hill tribe members lived [5]. The chosen area warrants attention due to its transportation inaccessibility, as it is situated approximately 20 km away from Maejan Hospital. In the absence of regular and dependable public transportation options, patients and their family members may have to depend on their own vehicles, motorcycles, or even walking, resulting in travel times ranging from half an hour to up to 4.30 hours. For the depression care model development and testing, we conducted the CBPR in Ban Lao-Fu Village in 2019. The CBPR contained two phases: understanding the current situation of depression and care and model development.

### 2.2. The Operational Definition

Village health volunteers (VHVs) are appointed by the Ministry of Public Health (Thailand) and play a crucial role in healthcare development within communities. They receive training and supervision from primary and secondary healthcare staff to effectively provide care and knowledge to the people in their community. The microlevel of the ICCC framework is defined as building blocks at the patient and family interaction level. The mesolevel of the ICCC framework is defined as building blocks for the healthcare organization and the community. The macrolevel of the ICCC framework is defined as building blocks for favorable policies and the environment.

### 2.3. Setting and Samples

In Ban Lao-Fu Village, there were four groups (Akha, Mien, Lisu, and Lahu) of 2,402 people from 575 families. Each group had its own language and culture. The village had four Christian churches, one Thai primary school, two Chinese language schools, and a health promotion hospital as a primary healthcare centre at the subdistrict level. Among the 601 Ban Lao-Fu people aged 40 years and older, approximately 85.0% and 50.0% had no formal education and insufficient income, respectively. Furthermore, 52.0% were Buddhists and ancestor worshipers, and the rest were Christians. Ban Lao-Fu Village was purposely selected due to its high depression prevalence. Among those aged 40 years and older, 39.1% had depression [5]. Among these cases, 75.74% (*n* = 178) were diagnosed with mild depression, 17.88% (*n* = 42) with moderate depression, and 6.38% (*n* = 15) with severe depression [5]. Draft local procedures have been recommended for each group, including (i) enhancing participation in community events for individuals with mild depression, (ii) providing psychoeducation for those with moderate depression, and (iii) transferring individuals with severe depression to a secondary care hospital.

Based on data from the quantitative survey, ICCC framework, and family-community participation, we obtained qualitative data, developed the model, and evaluated it in the same Ban Lao-Fu Village community. Forty-five people were involved in the model development. Sixty-five people voluntarily participated in the model testing (as described in Implementation and Data Collection). Participants included 7 community leaders, 4 heads of people from the hill tribes, 2 healthcare personnel, 2 local administrative staff, 12 patients, 10 family members, and 28 village health volunteers (VHVs) who lived in this Ban Lao-Fu hill tribe village.

### 2.4. Ethical Considerations

Ethics approval for this study was granted by the Human Research Ethics Committee at Chiangrai Provincial Public Health Organization (reference: CRPPHO 6/2562, January 7, 2019). All participants were informed about the study and signed (either written or verbally) an informed consent to participate. They also verbally agreed to audio recording for the interviews, focus groups, and program participation. Their rights as research participants, based on the principles of the Declaration of Helsinki, were protected.

### 2.5. Research Instruments

A researcher-developed questionnaire assessing VHVs' knowledge, roles, and practice in depression care was used. The questionnaire based on the literature review contained 9 questions asking personal information, 10 yes/no questions assessing knowledge, 10 Likert-scale questions assessing attitudes, 10 frequency-rating-scale questions for the role practice as VHVs, and 5 scenario open-ended questions for practice skills. The validity, represented by the IOC index, and the reliability, represented by Cronbach's alpha coefficient, of the instrument for assessing VHVs' knowledge were found to be 0.88 and 0.83, respectively. Additionally, the validity, as indicated by the IOC index, and the reliability, as indicated by Cronbach's alpha coefficient, of the questionnaire for assessing VHVs' roles and practices were 0.86 and 0.85, respectively. In the qualitative data collection, a semistructured interview guide with open-ended questions was used to understand depression at each level (micro, meso, and macro) of the ICCC framework. Details of the interview guide are described below.

Understanding the current patient care for individuals from hill tribes with depression involves a sequential approach of look, think, and act. Qualitative data were collected at all levels.

#### 2.5.1. Microlevel: Building Blocks at the Patient and Family Interaction Level

We wanted to understand the problems and needs of patients with depression and their families. We conducted in-depth interviews with 10 people with depression and 7 relatives. Inclusion criteria were participants with moderate depression (Patient Health Questionnaire 9-item (PHQ-9) score of ≥10), who resided in the community for ≥6 months, understood Thai/their language, and voluntarily participated. A semistructured interview guideline with five open-ended questions was used to explore the current problems and participants' needs for the depression care. For example, what do you think about depression care for people from the hill tribes in your community? When people from hill tribes have depression? How do they/family/community manage and care for depression? The instrument and a description of the intended content of each question were shared with three subject matter experts (SMEs). SMEs scored each question based on the alignment of the question with the content description. The instrument showed good content validity with an item objective congruence (IOC) index was 0.81.

#### 2.5.2. Mesolevel: Building Blocks for the Healthcare Organization and the Community

We assessed the community's needs and problems in providing care for patients with depression. We collected data using a focus group and in-depth interviews.

- *In-depth Interview.* We interviewed seven healthcare personnel from the mental healthcare centres of Chiangrai Prachanukroh Hospital, Maejan Hospital, and Ban Lao-Fu primary healthcare centre, and one NGO, and one Christian leader in the hill tribe community. A semistructured interview guide with five questions was used. For example, what is current depression care/management for people from the hill tribes?
- *Focus Group.* We collected data using a focus group of 15 stakeholders, which included subdistrict administrative organization staff, heads of communities, and village health volunteers (VHVs). A semistructured guide with five open-ended questions was applied. For example, can you provide depression care to people from the hill tribes? What supports do you need in taking care of people from the hill tribes with depression?

The instruments at the mesolevel were validated by three SMEs using the same process described above, and the IOC was 0.87.

#### 2.5.3. Macrolevel: Building Blocks for a Favorable Policy and Environment

We assessed the current depression care policy. We conducted in-depth interviews with four people from four stakeholder groups (mental health providers from the mental healthcare centres of Chiangrai Prachanukroh Hospital, Maejan Hospital, Ban Lao-Fu primary healthcare, and subdistrict administrative organization staff). A semistructured interview guide with five open-ended questions was used to explore the health policy that supported the current care for people from the hill tribes with depression. Examples of questions included the following: What are policies for depression care for people from the hill tribes? Are there enough healthcare personnel to provide care for people from the hill tribes with depression? Three SMEs validated this instrument using the process described above, and the IOC was 0.81.

After data collection, we analyzed and synthesized data on current depression problems, strengths, and limitations of the current depression caring system using content analysis.

Based on a review of the data collected, the study team developed a draft of the depression care model for people from hill tribes with depression including the proposed interventions. The draft was shared with five SMEs, who were mental health and psychiatric community specialists and stakeholders to review. They provided additional suggestions for content and evaluated the overall validity of the proposed interventions for each level. Overall, the IOC was 0.83.

After we revised our draft model, we conducted a pilot test by implementing this draft model among people from hill tribes with depression, their families, and community members of Ban Lao-Fu hill tribe community at the Ban Lao-Fu primary healthcare centre. We analyzed and synthesized data from the pilot test using content analysis to identify the themes (e.g., depression problems, barriers, strengths, and limitations of pilot testing). We summarized data, revised the depression care model, and employed seven SMEs for connoisseurship validation.

### 2.6. Data Analysis

Content analysis was used for qualitative data. The Wilcoxon signed-rank test was applied for quantitative data regarding the comparison of VHVs' knowledge and roles in depression care between pre- and postintervention of the program. The Wilcoxon signed-rank test was applied due to the nonnormal distribution of data and the small sample size.

### 2.7. Implementation and Data Collection

**Figure 1:**
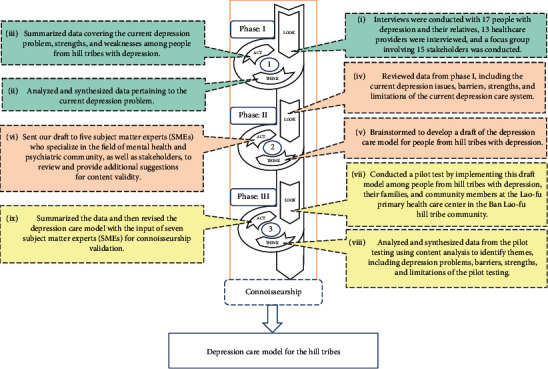
Developing and evaluating the depression care model for the hill tribes.

The model development contained three phases using the Stringer concept [21], which consisted of the look, think, and act strategies as spiral steps, as illustrated in Figure 1. In all phases of model development, we employed four different language translators. The patients, their families, and community members received money as their daily income subsidy for their participation.

## 3. Results

We used the ICCC as the theoretical framework and CBPR as the methodological process to develop a depression care model. The ICCC consisted of three levels, the micro-, meso-, and macrolevels, which corresponded to building blocks at the patient and family interaction level, for the healthcare organization and the community, and a favorable policy and environment, respectively. These three levels have been described in further detail in Methods and Results. The ICCC was used to identify the problems and develop, implement, and evaluate the model at all three levels.

### 3.1. Understanding the Current Patient Care for People from Hill Tribes with Depression

#### 3.1.1. Microlevel

Most people did not understand depression, especially its definition, causes, and symptoms. They did not define depression as an emotional disorder that needed to be cured. Instead, they defined it as overthinking, suffering, isolation, laziness, headache, insomnia, and parasomnia. For them, it meant a *Phi-Ba* or “mad ghost,” which was an evil spirit that resided in a person with depression or mental health problems. Therefore, it was associated with stigma for individuals and families to have mental health problems. Hence, it was difficult for them to understand and accept. Furthermore, they also had limitations in accessing the healthcare centre due to no Thai identification documents, communication barriers, travel obstacles, and economic issues.

#### 3.1.2. Mesolevel

We identified depressed cases when they came to the primary healthcare centre for care and treatment of their physical illness. The healthcare centre did not have an explicit system for direct depression screening. Additionally, there was no mental health clinic or specific psychosocial treatment for patients with mild and moderate depression. However, in severely depressed cases, the primary healthcare providers referred them to a specific healthcare centre/tertiary hospital. Primary healthcare providers were only responsible for home visits and cooperation with the relevant health resources.

The strengths of the current depression care in the Ban Lao-Fu community were found to be as follows. The stakeholders were willing to solve the communities' depression problems and help with universal health insurance and other issues. Notably, most community members loved their families and community and supported each other. They always gathered on Sunday at the church for choir singing and to worship God. Participating in these services and cultural-related ceremonies provided the community with a sense of unity and mental support for each other. Moreover, the community members believed in and followed the heads of their communities. Through village loudspeaker broadcasting, they received information, news, and announcements from their community leaders (village heads).

The following barriers should be noted regarding the current depression care in the Ban Lao-Fu community. We found that VHVs and community members had low self-awareness and knowledge regarding mental health issues. They defined depression as an ordinary situation that could occur in everyone. Interestingly, they did not think that this problem required help from healthcare services. Since they were poor and had a low education level, they, similar to other community members, mostly paid more attention to their basic needs compared to health issues. In addition, the VHVs' competency in caring for people from hill tribes with depression was low, especially regarding screening and basic counselling skills. Moreover, some could not speak the Thai language. Although the primary healthcare service had translators, some patients with depression did not trust and talk with the translators and health providers since depression was considered a sensitive and confidential issue. Besides these barriers, personal obstacles (for example, personality and perception) and their culture contributed to inaccessibility to depression care and treatment. Concerning the culture of self-care practice, when they were sick, most prayed to their ancestor ghosts (*Phi Banpaburut*) to get better or obtain protection. Therefore, almost every household had an ancestor worship altar (*Hing Banpaburut*). They also believed that one had a physical or mental health problem due to *Phi-Ba* or a mad ghost that replaced one's good spirit (“Kwan”). This Phi-Ba resided in an ill person who had a weak Kwan. To chase away this evil spirit and bring back a good spirit, they had to do a ritual ceremony of “Reak Kwan,” which was to call a good spirit back to that person. This practice and belief had both positive and negative impacts on an individual's health. Negatively, they did not seek help and care from professional healthcare providers and services. Positively, the practice and ceremony provided them with mental support from the ceremony leaders and people who participated. It also brought hope and a positive mind of getting better to the patients and their families.

Taken together, the depression management at the mesolevel should be described as follows. Most individuals from hill tribes with depression utilized emotion-focused coping skills to deal with their problems. However, they lacked sufficient appraisal-focused coping skills to address their issues directly. Additionally, there was no surveillance program in place to regularly screen and monitor patients with depression in the community.

#### 3.1.3. Macrolevel

Although the Thai government supported the healthcare teams in screening individuals' mental health and referring cases by following the public health guideline, it was not suitable for the hill tribes' context (communication problems and culture/beliefs). The mental health policy was appropriately established for the general population but was not suitable for the hill tribes due to communication problems, travel obstacles, and economic issues.

### 3.2. Reviewing and Synthesizing Information for the Development of a Caring Model

#### 3.2.1. Microlevel: Building Blocks at the Patient and Family Interaction Level

According to the ICCC framework [11], patients and their families, community partners, and healthcare teams achieved positive outcomes for chronic conditions only when they worked together, and patients and families were informed regarding their health conditions, motivated to change and maintain them, and prepared to learn behavioural skills. Therefore, our goal was to encourage patients with depression and their families to participate in activities with the community and VHVs. Families, VHVs, and community members could support patients with depression to increase their self-esteem and manage their problems themselves.

#### 3.2.2. Mesolevel: Building Blocks for the Healthcare Organization and Community

Concerning the ICCC framework, healthcare organizations and leaders in the community could raise awareness and reduce stigma. In addition, the community could achieve better outcomes through community leadership, support, resource mobilization, coordination, and complimentary services. Hence, our goal was to raise awareness and increase the competency of VHVs as community members and healthcare leaders in the community for dealing with patients with depression. The competency preparation consisted of providing health information on how to care for patients with depression and preparing effective health strategies to increase self-awareness and reduce stigma. We also aimed to support and cooperate with healthcare organizations and other community resources for depression screening, prevention, and treatment.

#### 3.2.3. Macrolevel: Building Blocks for a Favorable Policy Environment

At this level, the policy environment to support chronic healthcare in the community included leadership and advocacy, policy integration, consistent financial support, human resource development and allocation, legislative framework, and partnership strengthening. Consequently, our goal was to provide and create a healthcare policy that supported healthcare, related organizations, and the community in caring for patients with depression. These organizations included subdistrict administrative organizations, administrative staff, heads of the hill tribes, and community leaders. The information related to communication problems, travel obstacles, and economic issues has been discussed among subdistrict administration staff and members of the municipal council. Some issues, such as the lack of budget for managing travel obstacles, providing convenient public transportation, recruiting more staff, and establishing efficient ways of communicating with community members, have emerged as needs for the organization's policy. The policy changes included strategies to increase access to healthcare centres for patients with depression even without the Thai identification documents required to receive universal healthcare. These patients have access to social welfare benefits and services such as routine transportation from the villages to the hospitals, the home visit program conducted by well-trained healthcare staff, and funding offered by the local government to support the patients during their treatment process. They helped and coordinated with patients, their families, the community, and other related organizations to facilitate the hill tribes' access to mental health services, even without Thai identification documents.

### 3.3. Developing a Caring Model for the Hill Tribes with Depression

After we reviewed and synthesized the data, we established the goals of each level regarding the ICCC framework and developed the depression care model.

To reach all the goals, we created a new care model for people from hill tribes with depression. We named it “SMILE” and implemented it through activities that aimed to inform, motivate, and prepare patients, families, and the community for depression care. The model is aimed at increasing patients' self-awareness and self-esteem and involving the community and stakeholders in the depression care model. Each letter of the “SMILE” model was defined as follows:

S: this stands for stakeholders' readiness to care for people from hill tribes with depression.

M: this stands for motivation of people from hill tribes with depression to change their behaviours and of their family and community in depression care. The motivation was divided into two parts: external and internal motivations. The external motivation was the power of stakeholders that supported the patients with depression to receive treatment. The internal motivation was the power of mind among the stakeholders and people from hill tribes with depression for enhancing the willingness to engage in care. Internal and external motivations were developed through the motivation strategies of “we think, we can do” to improve self-esteem among the patients and the self-confidence of the families and community members, particularly VHVs in depression care [22].

I: this stands for interpersonal relationships, such as the relationship between patients, their family members or relatives, VHVs, and community leaders, or the relationship between patients and their families and healthcare providers. Interpersonal relationships were built and promoted through all activities and programs in the model's development and implementation.

L: this stands for “life and community asset” of the hill tribes. People from the hill tribes loved their families and community. They viewed their lives in positive ways and always had hope for the future. They believed in and followed their tribe's heads. The community had four churches that provided them with positive mental and spiritual support.

**Figure 2:**
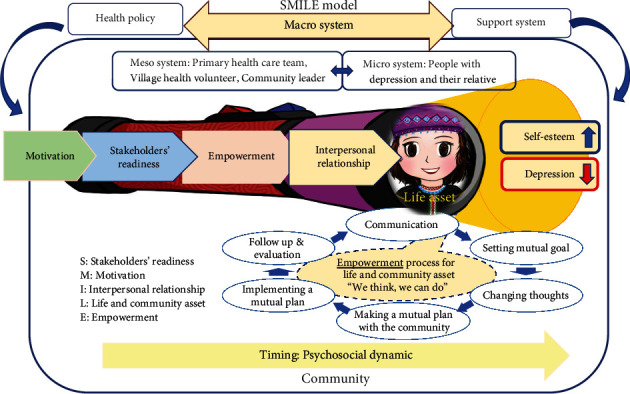
Diagram of the depression care model for people from hill tribes with depression based on family and community participation. The “SMILE” model was approved through a connoisseurship validation. Its IOC was 0.82. The implementation of this model can be driven through activities that aim to inform, motivate, and prepare patients, families, and the community for depression care. At the mesolevel, the lesson learned from depression care at the healthcare centre, especially in the Ban Lao-Fu community, including its current situation, strengths, and barriers, was described and improved. The knowledge level about depression and basic counselling in both patients and their relatives was measured and developed (the microlevel). At the macrolevel, a favorable policy environment to support chronic healthcare, such as leadership and advocacy, the integration of policies, consistent financial support, the development and allocation of human resources, a legislative framework, and the strengthening of partnerships, was developed.

E: this stands for “empowerment.” To achieve the goals of model implementation, the stakeholders, such as healthcare providers, needed to empower the patients, their families, VHVs, and community members to take care of the patients with depression, based on their culture and contexts (Figure 2).

#### 3.3.1. Concept of the “SMILE” Model

We developed this model based on humanistic theory, which emphasizes that people have motives and rational beliefs and that they can be socialized. Everyone could choose their way to improve competency and achieve their goal if they had enough freedom to decide and environmental support. All these led them to be a full potential person with self-actualization. Roger classified the human-self into three characteristics [23]: (1) perceived self, which was defined as how a person saw the self and others saw them; (2) real self or authentic self, which was how a person truly was; and (3) ideal self, which was how a person would like to be. To decrease depression, patients, their families, the community, healthcare providers, and other stakeholders should work together to help the patients recognize their perceived, real, and ideal selves and be aware of their problems. For a flow diagram of the depression care model based on family and community participation, see Figure 2.

### 3.4. Training VHVs in the “SMILE” Model

For VHVs, we conducted a three-day health education program, providing training to 28 VHVs. We also invited stakeholders, including healthcare providers from the healthcare centre and mental healthcare personnel, to participate in the training. The program included depression care, basic health counselling, and VHVs' roles in mental health problems. We involved a real patient in a counselling role play performed by a VHV and led by a psychologist. This patient shared with the community to overcome the stigma.

In all the activities, 15 stakeholders, including 7 community leaders, 4 heads of the hill tribes, 2 officers from the local administration organization of Ban Lao-Fu, and 2 healthcare providers, supported the implementation of SMILE model and the mental health policy, guided by the Ministry of Public Health. They helped and coordinated with patients, their families, the community, and other related organizations to facilitate the hill tribes' access to mental health services, even without Thai identification documents.

### 3.5. The Provided Training Program Improves Depression and Basic Counselling Knowledge of the VHVs

Of the 28 participants, 78.60% were female (see Table 1). The participants' ages ranged from 32 to 58 years old, with a mean age of 42.32 years (SD = 6.00). The majority (92.9%) of the participants were married. Most participants had a primary school education (82.10%), and 57.14% had sufficient income.

Among the 28 VHVs who attended the workshop, we found that they had more depression screening skills compared to before and could provide basic health counselling to the patients and families. We also used the Wilcoxon signed-rank test to assess the scores of knowledge relevant to depression and the roles of VHVs before and after the training. In accordance with Table 2, both scores were significantly increased (*p* < 0.001).

## 4. Discussion

In this present study, the depression care model for the hill tribes, named the “SMILE” model, which consists of stakeholders' readiness (S), external and internal motivations (M), interpersonal relationships (I), life and community assets (L), and empowerment (E), has been successfully developed for the first time. The obtained model was divided into three phases, including (i) understanding the current situation of depression and care, (ii) model development, and (iii) evaluation of its effectiveness using psychological and relevant outcomes.

We found that these voluntary healthcare providers had better skills for screening for mental health problems (for example, anxiety and depression) following the training. In addition, they could explicitly identify the difference between patients with depression and other psychiatric patients and communicate the essential health information about patients to mental health nurses at the primary healthcare centre. Furthermore, they had more basic counselling skills to support people from the hill tribes with depression. This training workshop for VHVs helped prepare them to provide care for people from hill tribes with depression. In training, the volunteers could share their thoughts and opinions with each other. It helped them gain knowledge and develop a positive attitude [24]. In low-resource settings, training village healthcare volunteers to conduct basic mental healthcare screening, monitoring, and counselling could prevent and minimize mental health problems of people in the community [25].

Several VHV, community leaders, and local administrative staff suggested that knowledge and information on depression care could be communicated through village loudspeaker broadcasting in the hill tribes' language by the heads of the community. This strategy could increase both awareness and acceptance of depression in the community. Therefore, the heads of communities, language translators, and church leaders should be trained and involved in the depression management program.

The academic research team, alongside patients, families, the community, and stakeholders, engaged in reflection and synthesis of key lessons learned from the model pilot. These lessons emphasize the significance of cultural sensitivity, as many individuals from hill tribes have heightened sensitivity and feelings of inferiority compared to native Thai people. For the depression care model to be effective, therapists, healthcare personnel, and stakeholders must possess three crucial qualities. Firstly, they should demonstrate a positive attitude towards patients with depression through consistent actions and words. Additionally, they must practice acceptance, refrain from judgment and criticism, and offer unconditional compassion. Lastly, an empathetic understanding is essential to foster success in implementing the model. It should be highlighted that the implementation of positive and effective communication skills between the academic research team and participants is one of the crucial factors in enhancing their enthusiasm for participating in the model pilot.

Additionally, stakeholders synthesized the empowerment process based on life and community assets, encompassing various crucial elements. Effective communication, especially within hill tribes where depression is a sensitive topic, played a significant role in helping individuals with depression understand themselves and raising awareness in the community [26]. Setting mutual goals among patients, families, and the community fostered an understanding of the benefits of depression care and management, along with enhancing their self-perception [27]. Encouraging individuals with depression to reevaluate their thoughts and beliefs contributed to increased self-confidence [28]. Collaboratively developing and implementing depression care plans within the community created a sense of acceptance and belonging for patients [29, 30], leading to heightened self-esteem [31]. The follow-up and evaluation step ensured the model's effectiveness, allowing revisions based on family and community input [32]. While the ICCC framework-based model utilizing family and community participation proved effective in caring for hill tribes with depression, some limitations were noted in its development and implementation.

According to the ICCC framework, building blocks for a favorable policy environment from every involved party is essential for successful chronic disease management [12]. However, our SMILE model focused mainly on patients, families, and the community. There was no intervention for the stakeholders, such as healthcare providers and authorities together with the community, for establishing and implementing the mental healthcare policy specific for the hill tribes with depression. Nevertheless, most healthcare providers provide mental healthcare in line with the policies already established by the Ministry of Health. These policies were not specific for the hill tribes, who are vulnerable groups with social disparities and language barriers that affect their ability to access healthcare [33].

In family- and community-based participatory research for chronic care management, the family, community, and stakeholders should participate in all research steps, from data collection, model development, implementation, and outcome evaluation. However, due to the COVID-19 pandemic, it was challenging to involve every party in every study step. Most healthcare providers and authorities focused on COVID-19 prevention and management at the beginning of the pandemic. Like other CBPR studies, it is difficult to achieve full participation from all parties involved in the research project at every step of the CBPR, given the time and energy required [34].

Despite the successful improvement of participants' knowledge and basic screening and counselling skills by the SMILE model, certain obstacles and limitations must be addressed. Firstly, the development of this model was constructed based on the situation within one primary healthcare unit. Although the study area encompasses four ethnic groups, Chiang Rai has six majority ethnic groups [10], and Thailand has 56 majority ethnic groups in 67 provinces [35]. While the effectiveness of the SMILE model has been proven in Ban Lao-Fu Village [36], it must be validated in additional ethnic groups outside the study site. Secondly, this model was developed based on the hill tribes who are fluent in reading and speaking the Thai language. Therefore, further development of the SMILE model should also focus on those hill tribes who do not speak the Thai language. Thirdly, as this model requires on-site participation, it can be a significant burden and an extremely stressful experience for people living in poverty.

## 5. Conclusions

The “SMILE model” is a depression care model developed based on family and community participation for people from hill tribes with depression. It is a well-formulated and framework-based model that incorporates fundamental components related to the patient, family, healthcare organization, community, and policy levels. It has the potential to support patients with depression and their family members in the hill tribe communities.

## Figures and Tables

**Table 1:** Participants in basic screening and counselling for village health volunteers (*n* = 28).

Characteristics of participants	*N* or mean	% or SD
Gender (male/female)	6/22	21.40/78.6
Age (in years)	42.32 (min = 32/max = 58)	6.00
Marital status		
Single	2	7.1
Married	26	92.9
Education		
Illiterate	3	10.70
Primary school	23	82.10
Secondary	1	3.6
Diploma	1	3.6
Sufficient income (yes/no)	16/12	57.14/42.86

**Table 2:** Change in participants' knowledge and basic counselling and depression screening skills before and after the training.

	Knowledge	Basic screening and counselling
Median score (*n* = 28)		
Before training	4	3
After training	8	7
Median change in percentage points (delta) (*n* = 28)	4	4
Signed-rank test statistic (*Z*)	-4.659	-4.194
*p* value	<0.001	<0.001

## Data Availability

Due to the ethical concern with participants' data and privacy, the datasets obtained and/or analyzed during the current study are not publicly available. The information and materials discussed in the manuscript are, nevertheless, accessible upon the relevant author's justifiable request.
